# Formation‐Stage Interfacial Regulation of δ‐MnO_2_ Enables Stable and Reversible Aqueous Zn‐Ion Storage

**DOI:** 10.1002/advs.78465

**Published:** 2026-11-04

**Authors:** Fanghua Liang, Ling Wang, Junpeng Xiong, Chunqing Niu, Atsuya Kawai, Wei Zhang, Chunhong Zhu, Azeem Ullah, Mayakrishnan Gopiraman, Mingzheng Ge, Ick Soo Kim

**Affiliations:** ^1^ Nano Fusion Technology Research Lab Institute For Fiber Engineering and Science (IFES) Research Cluster for Social Implementation Shinshu University Ueda Nagano Japan; ^2^ School of Textile and Clothing Nantong University Nantong People's Republic of China

**Keywords:** aqueous zinc‐ion battery, Fe precursor encoding, interfacial electronic regulation, Mn dissolution, Mn─O framework, δ‐MnO_2_ nanosheets

## Abstract

Layered δ‐MnO_2_ is a promising cathode for aqueous zinc‐ion batteries, yet its practical application remains hindered by the coupled challenges of sluggish Zn^2+^ storage kinetics and persistent Mn dissolution. Here, we develop a precursor‐embedded Fe interfacial regulation strategy to modulate the nucleation and growth of δ‐MnO_2_ on porous carbon nanofibers (δ‐MnO_2_@Fe‐PCNFs). Rather than being dominated by homogeneous bulk substitution, the introduced Fe is predominantly present as low‐content, highly dispersed surface‐/interface‐associated species established during δ‐MnO_2_ formation, where it regulates the local Mn─O environment and consequently modulates nanosheet growth and the accessibility of the reaction interface. The Fe‐regulated δ‐MnO_2_ framework exhibits improved kinetic accessibility and suppresses Mn dissolution without altering its intrinsic Mn‐based redox chemistry. Consequently, the δ‐MnO_2_@Fe‐PCNFs electrode delivers an initial capacity of 409.29 mAh g^−1^ at 0.2 A g^−1^ and excellent long‑term cycling stability, maintaining a capacity of 243.64 mAh g^−1^ at 1 A g^−1^ after 1000 cycles and excellent capacity retention of 81.1% after 3000 cycles at 5 A g^−1^. This work highlights formation‐stage interfacial regulation as an effective strategy to couple reaction accessibility with structural durability in Mn‐based aqueous zinc‐ion battery cathodes.

## Graphical abstract

Precursor‐embedded Fe regulates the nucleation and growth of δ‐MnO_2_, enabling a more accessible and stable Zn‐storage interface with accelerated kinetics and suppressed Mn dissolution, while preserving the intrinsic Mn redox chemistry.

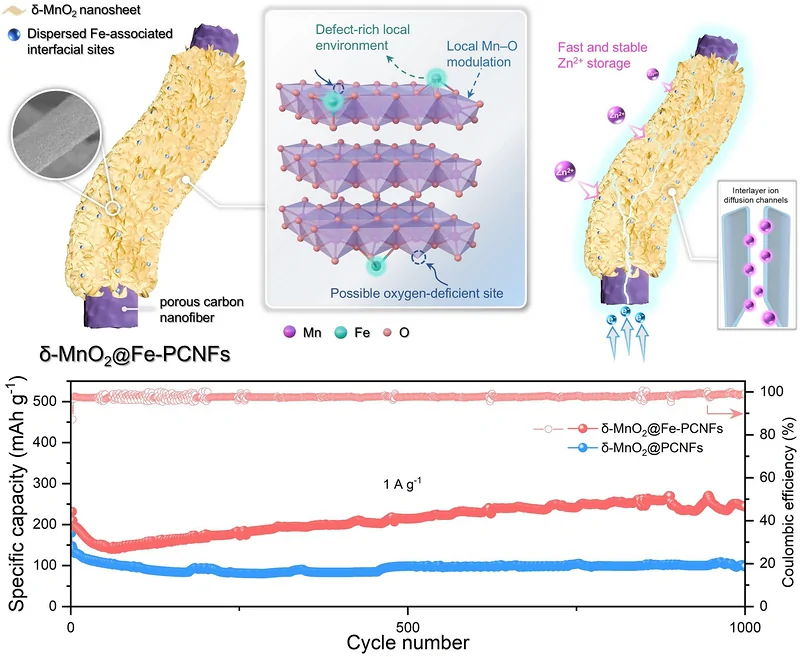

## 1 Introduction

Aqueous zinc‐ion batteries (AZIBs) have been widely recognized as promising energy storage systems owing to their intrinsic safety, environmental compatibility, low Zn redox potential of −0.76 V versus the standard hydrogen electrode, and abundant zinc resources [1, 2, 3, 4, 5, 6, 7]. Among various cathode candidates, layered δ‐MnO_2_ is particularly attractive because of its high theoretical capacity and large interlayer spacing of approximately 0.7 nm, which facilitates Zn^2+^ insertion and extraction [8, 9, 10, 11]. Nevertheless, its practical application is limited by two coupled challenges. The Zn^2+^/H^+^ co‐storage process is kinetically sluggish because of slow charge transfer and ion diffusion [12, 13, 14, 15, 16]. Meanwhile, continuous interfacial charge accumulation during cycling promotes Mn dissolution, destabilizes the local reaction environment and MnO_2_ framework, and intensifies parasitic reactions and polarization [17, 18, 19, 20].

To address the kinetic limitation, intrinsic activity regulation strategies such as bulk doping and defect engineering have been widely explored [21, 22, 23, 24, 25, 26]. Oxygen vacancy engineering has been shown to enhance Zn storage behavior by tailoring defect concentrations and activating local electrochemical sites [27, 28, 29, 30, 31]. For example, Wang et al. regulated oxygen vacancy levels in flower‐like δ‐MnO_2_ and achieved high capacity and high‐rate response, confirming the effectiveness of defect engineering in activating intrinsic reaction kinetics [32]. Similarly, lattice doping with heteroatoms such as Co and Fe has been widely adopted to modulate the MnO_2_ framework, wherein local coordination and orbital coupling modify the electronic structure and defect states of the host lattice, thereby improving redox kinetics and intrinsic conductivity [33, 34, 35, 36]. However, these intrinsic modulation approaches primarily target the reactivity within the δ‐MnO_2_ bulk, whereas the interfacial instability arising from Mn dissolution and its cumulative effects over prolonged cycling often remain inadequately addressed [37, 38, 39]. To mitigate Mn dissolution and parasitic reaction accumulation, surface coating and interfacial stabilization strategies have been introduced into MnO_2_ cathode design [40, 41, 42, 43]. Ding et al. used amino acid molecules to construct a stabilizing layer on MnO_2_, effectively suppressing Mn dissolution and interfacial degradation [44]. However, this strategy mainly stabilizes an already formed reaction interface rather than shaping MnO_2_ nucleation and the local reaction environment during material formation [45, 46, 47]. Therefore, existing strategies focus on modifying the MnO_2_ host lattice or stabilizing its already formed interfaces, whereas the role of precursor chemistry in directing MnO_2_ nucleation, growth, and subsequent electrochemical interface formation remains less systematically explored. How the local interfacial chemistry established at the precursor stage participates in the nucleation and growth of δ‐MnO_2_, and further determines the final nanosheet structure and electrochemical reaction interface, remains to be clarified. Thus, direct regulation of the growth interface during material formation offers a strategy distinct from conventional bulk doping and post‐synthetic surface modification, thereby linking of material formation to subsequent electrochemical behavior. Notably, Fe is a candid for interfacial regulation owing to its strong oxygen affinity and potential to induce local electronic redistribution within manganese oxide frameworks [33, 36].

Here, we develop a formation‐stage interfacial regulation strategy for δ‐MnO_2_, distinct from conventional high‐concentration bulk substitution or post‐synthetic protective‐layer construction. By introducing a small amount of Fe during precursor processing, an Fe‐regulated δ‐MnO_2_/porous carbon nanofiber composite cathode (δ‐MnO_2_@Fe‐PCNFs) is prepared by electrospinning followed by oxidation. During the subsequent oxidation process, low‐content Fe regulates δ‐MnO_2_ nanosheet nucleation, surface growth, and the local Mn─O environment, thereby coupling growth‐interface control with the evolution of the final reaction interface. As a result, the δ‐MnO_2_ framework delivers improved Zn^2+^ storage kinetics and reduced Mn dissolution without changing its intrinsic Mn‐based redox chemistry. Consequently, the δ‐MnO_2_@Fe‐PCNFs exhibit robust electrochemical performance, delivering an initial discharge capacity of 409.29 mAh g^−1^ at 0.2 A g^−1^, retaining 243.64 mAh g^−1^ after 1000 cycles at 1.0 A g^−1^, and maintaining a capacity retention of 81.1% after 3000 cycles at 5 A g^−1^. More broadly, this work establishes a route to couple how a layered oxide cathode forms with how it subsequently reacts, offering a new design principle for synchronizing kinetic accessibility and structural durability in aqueous energy‐storage materials.

## 2 Results and Discussion

To simultaneously address the sluggish reaction kinetics and Mn dissolution‐induced structural instability of δ‐MnO_2_, we propose a precursor‐embedded interfacial regulation strategy. Figure 1 schematically illustrates the synthesis route, structural model, and Zn storage pathway of the Fe‐regulated δ‐MnO_2_@PCNFs cathode (δ‐MnO_2_@Fe‐PCNFs). In this design, Mn precursors together with a small amount of Fe species are introduced during electrospinning to form embedded active sites within the developing nanofiber framework. After carbonization, Mn‐ and Fe‐containing porous carbon nanofibers (Mn, Fe@PCNFs) are obtained. Subsequent oxidation in KMnO_4_ solution induces the in situ growth of δ‐MnO_2_ nanosheets on the surface of the porous carbon nanofibers, leading to the formation of the δ‐MnO_2_@Fe‐PCNFs composite. The embedded Fe species regulate the growth of δ‐MnO_2_ nanosheets. Rather than being dominated by homogeneous bulk lattice substitution or segregated Fe‐rich crystalline phases, Fe is predominantly present as low‐content, highly dispersed oxygen‐coordinated species associated with the surface/near‐surface and interfacial regions of δ‐MnO_2_. Such Fe‐associated local coordination perturbs the Mn─O electronic and charge‐compensation environment, promoting local electronic redistribution and favoring the formation or stabilization of oxygen‐vacancy‐related defects. Based on this structural model, the porous carbon nanofiber network provides efficient electron‐transport pathways, while Fe regulation modifies the nucleation and growth behavior of δ‐MnO_2_ nanosheets and improves the accessibility of interfacial Zn^2+^ storage sites. The resulting nanosheet architecture further reduces the effective ion‐transport length and facilitates Zn^2+^ access to the reaction interface. This synergistic regulation of the conductive scaffold, local Mn─O environment, and nanosheet architecture contributes to both enhanced interfacial reaction kinetics and improved structural reversibility of the δ‐MnO_2_ framework.

**FIGURE 1:**
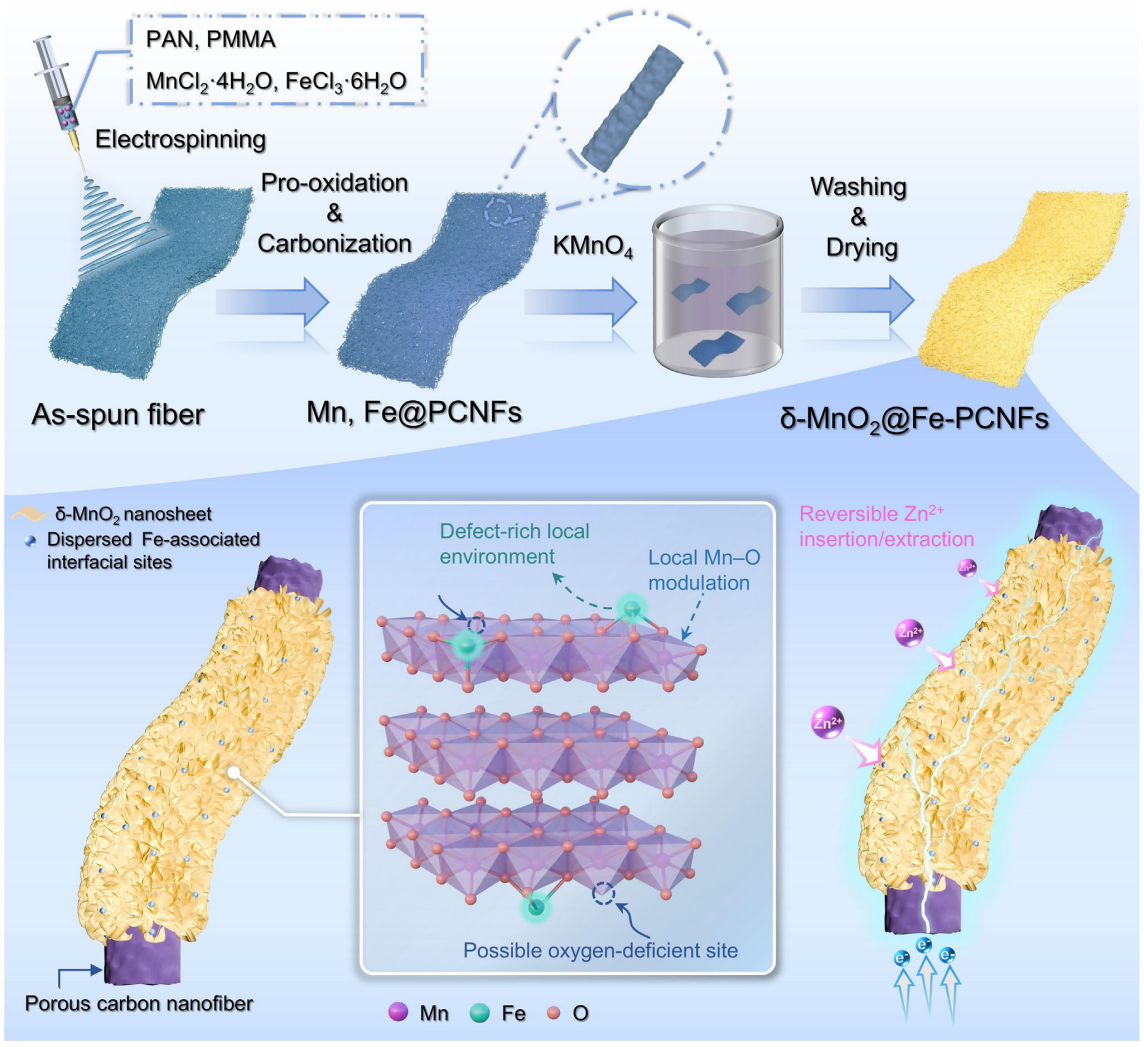
Schematic illustration of the synthesis route and proposed interfacial regulation mechanism of Fe‐regulated δ‐MnO_2_@PCNFs. δ‐MnO_2_ nanosheets are grown on porous carbon nanofibers, while Fe is represented as dispersed Fe‐associated interfacial sites rather than a uniform bulk dopant or an independent crystalline phase. A representative Fe─O_3_ motif and a neighboring defect‐rich oxygen region are used to illustrate the Fe‐associated local Mn─O perturbation. The porous carbon nanofiber framework provides electron transport pathways, while the δ‐MnO_2_ nanosheet arrays shorten Zn^2+^ diffusion pathways and promote a more accessible interface‐dominated Zn‐storage process.

To gain atomic‐level insight into the underlying role of Fe interfacial modulation in regulating the electrochemical behavior of δ‐MnO_2_, density functional theory (DFT) calculations were performed to elucidate the electronic structure, defect chemistry, and Zn adsorption characteristics. To directly distinguish the effects of Fe location, three parallel models were constructed using consistent computational settings: pristine δ‐MnO_2_, a representative surface‐associated δ‐MnO_2_@Fe configuration, and a lattice‐Fe‐substituted δ‐MnO_2_ configuration (Fe_Mn_‐δ‐MnO_2_) (Figure S1). Considering that the experimental observations support a predominantly surface‐/interface‐associated Fe distribution but do not uniquely resolve its atomic coordination, the surface Fe─O─Mn‐like configuration was employed as a representative oxygen‐coordinated interfacial model rather than as a uniquely established Fe structure. The calculated band structures reveal band gaps of 2.19, 1.82, and 1.48 eV for pristine δ‐MnO_2_, Fe_Mn_‐δ‐MnO_2_, and δ‐MnO_2_@Fe, respectively (Figure S2). The progressive narrowing of the band gap indicates that Fe modifies the electronic structure of δ‐MnO_2_, with the interfacial configuration producing the most pronounced modulation. To further clarify the orbital origin of this electronic modulation, the corresponding total and projected density‐of‐states profiles (DOS/PDOS) profiles were analyzed (Figure 2a). Pristine δ‐MnO_2_ exhibits pronounced overlap between Mn 3d and O 2p states, reflecting the intrinsic Mn─O orbital hybridization of the host framework. Fe incorporation redistributes these states in a strongly location‐dependent manner. In Fe_Mn_‐δ‐MnO_2_, Fe 3d states contribute appreciably near the Fermi level together with O 2p states, indicating direct participation of lattice Fe in the host electronic structure. In contrast, the Fe 3d contribution near the Fermi level is relatively weak in δ‐MnO_2_@Fe, while overlap among Fe 3d, Mn 3d, O 2p states is mainly observed in the occupied valence region. This suggests that interfacial Fe regulates the Mn─O framework primarily through localized orbital coupling and electronic‐state redistribution rather than by introducing a dominant Fe‐derived conduction channel. Consistent with the calculated electronic‐structure evolution, ultraviolet‐visible (UV–vis) diffuse‐reflectance measurements show that the optical gap estimated form Tauc plots decreases from 2.23 to 1.68 eV after Fe incorporation (Figure S3). This trend is consistent with the DFT results, supporting the role of Fe in modulating the electronic structure of δ‐MnO_2_. The slight discrepancy between the calculated and experimental values may be associated with the tendency of conventional DFT calculations to underestimate band gaps. The effect of Fe location is further manifested in the Mn‐vacancy energetics. The Mn‐vacancy formation energies of pristine δ‐MnO_2_, Fe_Mn_‐δ‐MnO_2_, and δ‐MnO_2_@Fe are 12.63, 11.02, and 7.49 eV, respectively (Figure 2b). Although the Mn‐vacancy formation energies remain positive, the substantially lower value for δ‐MnO_2_@Fe indicates that interfacial Fe markedly reduces the energetic cost of Mn‐vacancy formation. The 3.53 eV difference in Mn‐vacancy formation energy between the δ‐MnO_2_@Fe and Fe_Mn_‐δ‐MnO_2_ configurations suggests that the exposed interfacial Fe exerts a much stronger perturbation on the local Mn─O coordination environment than conventional lattice Fe substitution, thereby endowing the interfacial region with higher structural adaptability toward defects. Zn adsorption also shows a pronounced dependence on Fe location. The adsorption energy becomes progressively more negative from −0.96 eV for pristine δ‐MnO_2_ to −1.47 eV for Fe_Mn_‐δ‐MnO_2_ and −1.76 eV for δ‐MnO_2_@Fe, demonstrating that both Fe configurations enhance the thermodynamic affinity toward Zn, with the interfacial configuration being the most favorable (Figure 2c). In the δ‐MnO_2_@Fe interfacial model, Zn initially placed adjacent to a surface O site spontaneously relaxes toward the exposed Fe‐top region during structural optimization. This relaxation indicates that δ‐MnO_2_@Fe does not merely perturb an existing O‐centered adsorption site, but reshapes the local adsorption‐energy landscape and establishes a distinct Fe‐associated adsorption environment. Charge density difference analysis further reveals this Fe location‐dependent adsorption behavior at the electronic level. For pristine δ‐MnO_2_, Zn adsorption primarily induces localized charge polarization around Zn and the adjacent surface O/Mn sites (Figure S4). Fe_Mn_‐δ‐MnO_2_ further modulates this charge response by altering the electronic environment of the host Mn─O framework, consistent with its substitutional structural feature. In contrast, the δ‐MnO_2_@Fe model exhibits a more pronounced and spatially continuous charge redistribution in the Zn─Fe─O─Mn region, indicating that the exposed Fe sites directly participate in the interface polarization induced by Zn adsorption, while this electronic response further extends to the surrounding Mn─O framework. Such distributed charge rearrangement suggests a coupled interfacial interaction rather than a purely isolated Zn─Fe or Zn─O interaction, providing an electronic‐structure basis for the stronger Zn adsorption calculated for δ‐MnO_2_@Fe. Overall, these results indicate that, compared with conventional lattice Fe substitution, the exposed interfacial Fe species enhance the Zn^2+^ adsorption and accessibility to interfacial active sites and promote faster interfacial charge transfer through the synergistic modulation of the local defect‑related environment and the Mn─O electronic structure.

**FIGURE 2:**
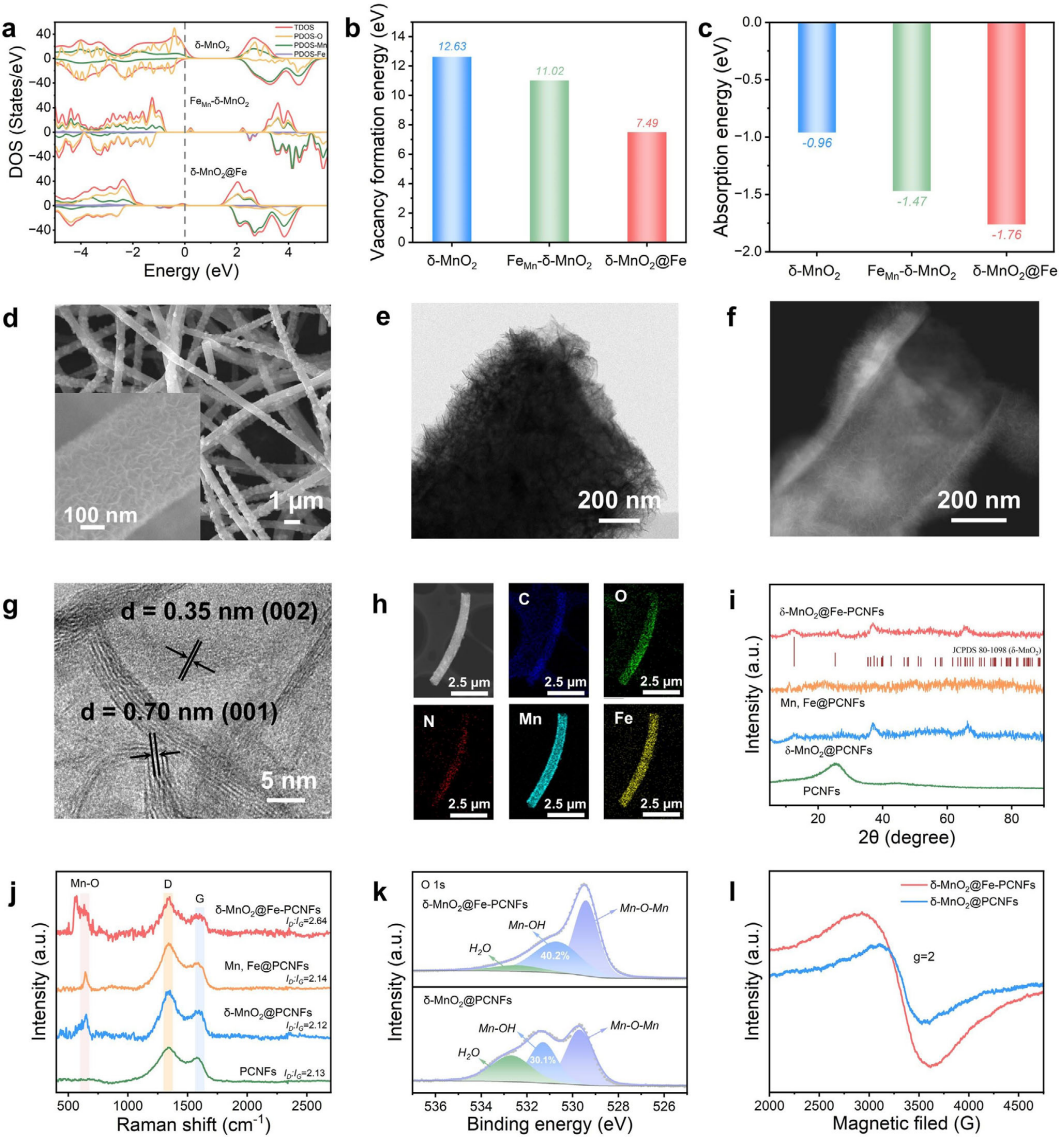
Theoretical and structural evidence for Fe‐regulated interfacial δ‐MnO_2_ formation. (a) Total density of states of δ‐MnO_2_, Fe_Mn_‐δ‐MnO_2_, and δ‐MnO_2_@Fe, as well as the projected density of states of O, Mn, and Fe atoms in δ‐MnO_2_ (001). (b) Mn surface vacancy formation energy and (c) Zn ion adsorption energy of δ‐MnO_2_, Fe_Mn_‐δ‐MnO_2_, and δ‐MnO_2_@Fe. (d) FE‐SEM, (e) TEM, (f) HAADF‐STEM, (g) HRTEM, and (h) STEM‐EDX mapping of the δ‐MnO_2_@Fe‐PCNFs. (i) XRD, (j) Raman of δ‐MnO_2_@Fe‐PCNFs, δ‐MnO_2_@PCNFs, Mn, Fe@PCNFs, and PCNFs. (k) High‐resolution O1s XPS spectra and (l) EPR spectrum of δ‐MnO_2_@Fe‐PCNFs and δ‐MnO_2_@PCNFs.

To examine the role of Fe regulation during the initial formation of δ‐MnO_2_ nanosheets, time‐dependent immersion experiments were carried out to track the morphological evolution of δ‐MnO_2_ during oxidative growth (Figure S5). δ‐MnO_2_ nanosheet arrays were observed on the Fe‐modified substrate within 30 min, whereas only sparse δ‐MnO_2_ nanosheets appeared on the unmodified substrate after 60 min. These results indicate that Fe promotes earlier δ‐MnO_2_ nanosheet formation and more uniform growth on the carbon nanofiber surface. Since Mn‑ and Fe‑related species already coexist in the precursor‑derived substrate before the KMnO_4_/ammonium persulfate treatment, such temporal and morphological differences suggest that Fe modifies the local oxidation and nucleation environment at the growth interface, thereby affecting the early formation pathway of δ‐MnO_2_ [48, 49, 50]. As shown in Figure 2d, compared with δ‐MnO_2_@PCNFs, the introduction of Fe enables the uniform in situ growth of high‐loading δ‐MnO_2_ nanosheets on the surface of porous carbon nanofibers, resulting in a structurally homogeneous δ‐MnO_2_@Fe‐PCNFs composite with an average fiber diameter of ∼470 nm. In contrast, δ‐MnO_2_@PCNFs exhibits a relatively disordered distribution of surface nanosheets (Figure S6a). This morphological difference further indicates that the embedded Fe species regulate the nucleation and growth interface of δ‐MnO_2_ during KMnO_4_ oxidation, thereby promoting uniform in situ nanosheet formation. Similarly, transmission electron microscopy (TEM) images reveal a marked structural difference between the two samples. Compared with the sparsely distributed nanosheets on δ‐MnO_2_@PCNFs, δ‐MnO_2_@Fe‐PCNFs exhibits a denser architecture with uniformly distributed nanosheets (Figure 2e and Figure S6b). Nitrogen adsorption‐desorption measurements further support this observation, showing a gradual decrease in specific surface area from PCNFs to δ‐MnO_2_@PCNFs and then to δ‐MnO_2_@Fe‐PCNFs (Figure S7 and Table S1). The reduced surface area indicates that the introduction of Fe enables δ‐MnO_2_ nanosheets to more effectively occupy the pore space of carbon nanofibers, confirming efficient δ‐MnO_2_ deposition and strong interfacial integration with the carbon framework. High‐resolution transmission electron microscopy (HRTEM) images of δ‐MnO_2_@Fe‐PCNFs show lattice spacings of 0.70 and 0.35 nm, corresponding to the (001) and (002) planes of δ‐MnO_2_ (Figure 2g). The selected area electron diffraction pattern of δ‐MnO_2_@Fe‐PCNFs further reveals a set of diffraction rings corresponding to interplanar spacings of 0.19 and 0.17 nm, which can be indexed to the (112) and (004) planes of δ‐MnO_2_, respectively (Figure S8). Scanning transmission electron microscopy (STEM) with energy‐dispersive X‐ray spectroscopy (EDX) mapping demonstrates the clear spatial distributions of C, N, O, and Mn within the composite architecture (Figure 2h). Notably, Mn exhibits a continuous distribution over the nanofiber region, consistent with the formation of δ‐MnO_2_ nanosheets. In contrast, Fe shows a more discrete and less continuous distribution than Mn. Meanwhile, the high‐angle annular dark‐field STEM (HAADF‐STEM) image shows that numerous ultrathin nanosheets are grown directly on the surface of the carbon nanofiber framework, forming intimate nanosheet–fiber contact interfaces. No obvious large high‐contrast particles are observed at the current observation scale (Figure 2f). Consistently, the STEM‐EDX elemental maps show that the spatial distributions of Mn and O closely match the nanosheet‐covered regions, confirming the distribution of the Mn─O phase (Figure S9). By contrast, the Fe signal is much weaker and is diffusely distributed within the Mn/O‐containing regions, without obvious localized Fe‐enriched hotspots. These spatial distributions indicate that the low‐content Fe species are highly dispersed and mainly associated with the δ‐MnO_2_‐rich regions. Therefore, EELS line‐scan analysis was further performed to examine the local spatial variation of the weak Fe signal between MnO_2_‐rich regions and exposed PCNF regions. As shown in Figure S10, the electron energy‐loss spectroscopy (EELS) line scan extends from the PCNF edge across the outer δ‐MnO_2_ region and further into the exposed PCNF region. When the electron beam passes through the nanosheet‐enriched region, the Mn L_3_/L_2_ signals at approximately 640–652 eV are markedly enhanced and then gradually decrease as the scan moves toward the bare PCNF region [51]. Meanwhile, the C K‐edge signal at approximately 293 eV becomes clearly intensified after entering the PCNF region. Because of the low Fe content, the Fe‐related EELS signals at approximately 711–722 eV are intrinsically weak. Nevertheless, weak Fe‐related signals are mainly detected in the δ‐MnO_2_‐associated and transitional regions with decreasing intensity toward the bare PCNF region. These results suggest that Fe exists in a low‐content and highly dispersed form and is preferentially spatially associated with the surface/near‐surface and local interfacial regions of δ‐MnO_2_, rather than forming large separated Fe‐enriched particles. In addition, samples with different Fe precursor loadings were compared, and both their morphological and optical responses exhibited a clear non‑monotonic Fe dependence, indicating that an appropriate amount of Fe favors the formation of a more uniform δ‐MnO_2_‑coated fiber structure (Figures S11 and S12).

X‐ray diffraction (XRD) patterns further corroborate this structural evolution (Figure 2i). After carbonization, Mn, Fe@PCNFs display broad, weak diffraction peaks, consistent with a predominantly amorphous structure, with no distinct peaks attributiable to crystalline δ‐MnO_2_. After immersion in KMnO_4_ solution, distinct diffraction peaks appear at 12.5°, 37.3°, and 65.6°, corresponding to the (001), (‐111), and (020) planes of δ‐MnO_2_ (JCPDS No. 80–1098) [52]. A broad peak at around 25.5° in PCNFs is attributed to graphitic carbon. Notably, no distinct diffraction peaks corresponding to independent Fe‐based crystalline phases are observed after Fe introduction. Instead, a notable peak shift is observed exclusively at the (002) reflection upon Fe introduction, suggesting that Fe more likely perturbs the local Mn─O environment rather than forming homogeneous bulk substitution. Quantitative fitting of the (001) reflection shows a shift from 12.56° to 12.36°, corresponding to a modest increase in the basal spacing from 7.04 to 7.15 Å (∼1.63%), whereas the higher‐angle δ‐MnO_2_‐related reflections exhibit only minor variations (<1%) (Figure S13 and Table S2). These limited structural changes indicate that the layered Mn─O framework is largely preserved and do not support extensive Fe intercalation as the dominant configuration. This interpretation is further supported by inductively coupled plasma optical emission spectroscopy (ICP‐OES) analysis, which reveals a low Fe content of approximately 3%, insufficient to enable lattice substitution or intercalation of Fe into the δ‐MnO_2_ framework (Table S3). Raman spectra exhibit a sharp peak at 646.5 cm^−1^, corresponding to the Mn─O stretching vibration of MnO_6_ octahedra (Figure 2j) [53]. Notably, δ‐MnO_2_@Fe‐PCNFs exhibit a stronger local Mn─O framework signal together with a broadened vibrational envelope. This result indicates that Fe introduction does not change the dominant phase of δ‐MnO_2_ but promotes complete and more effective in situ growth of δ‐MnO_2_ on the fiber surface. Meanwhile, Fe perturbs the local Mn─O coordination environment at the interface, giving rise to a more activated Mn─O framework that provides a flexible reaction space for electron and ion transport. Additional broad bands at ≈1350 and 1585 cm^−1^ correspond to the D band and G band, respectively. The higher I_D_/I_G_ ratio of δ‐MnO_2_@Fe‐PCNFs (2.64) than that of δ‐MnO_2_@PCNFs (2.12) indicates greater structural disorder in the carbon framework, potentially providing more sites for Zn‐ion transport and storage. This defect‐enriched carbon nanofiber surface facilitates the anchoring of Mn species and the subsequent in situ nucleation and uniform growth of δ‐MnO_2_ nanosheets. This result indicates that the embedded Fe species preconfigure a favorable interfacial foundation for δ‐MnO_2_ nanosheet growth. X‐ray photoelectron spectroscopy (XPS) was employed to analyze the surface chemical species of the samples. The survey spectra display representative peaks at ∼284, 529, 399, 642, 84, and 50 eV, corresponding to C 1s, N 1s, O 1s, Mn 2p, Mn 3s, and Mn 3p, respectively (Figure S14a) [8]. A distinct Fe 2p signal at ∼712 eV confirms the presence of Fe in δ‐MnO_2_@Fe‐PCNFs. The Mn 2p_3/2_ spectrum can be deconvoluted into Mn^3+^ and Mn^4+^ components. The increased Mn^3+^ fraction after Fe introduction suggests that Fe modulates the local MnO coordination environment and promotes defect‐related electronic reconstruction [54]. The altered Mn^3+^ distribution is more favorable for the generation of oxygen vacancies (Figure S14b). As shown in Figure 2k, the O 1s spectrum consists of lattice oxygen (Mn─O─Mn, ∼529.9 eV), hydroxyl‐/defect‐associated oxygen (Mn─OH, ∼531.5 eV), and adsorbed water/high‐binding‐energy surface oxygen species (H_2_O, ∼532.9 eV). Notably, the relative contribution of the hydroxyl‐/defect‐associated oxygen component reaches 40.2% in δ‐MnO_2_@Fe‐PCNFs, significantly higher than δ‐MnO_2_@PCNFs (30.1%). Meanwhile, the relative contribution of the high‐binding‐energy adsorbed species decreases after Fe introduction, indicating a redistribution of the surface oxygen chemical states toward a higher proportion of hydroxyl‐/defect‐associated oxygen environments. These results suggest that Fe regulation modifies the local oxygen coordination environment, which may contribute to enhanced interfacial reactivity. The Fe 2p spectrum can be fitted into Fe^2+^ (710.3 eV) and Fe^3+^ (712.5 eV) components, accompanied by a satellite peak at 717.4 eV, indicating that Fe mainly exists in an oxidized state rather than in a metallic state (Figure S14c) [55]. The mixed Fe^2+^/Fe^3+^ character is consistent with its interaction with the local Mn─O coordination environment. To further probe the defect‐related electronic states, electron paramagnetic resonance spectroscopy (EPR) measurements were conducted. Compared with δ‐MnO_2_@PCNFs, the δ‐MnO_2_@Fe‐PCNFs electrode exhibits a significantly stronger EPR signal with a g value close to 2.0, indicating enhanced defect‐related paramagnetic centers and unpaired‐electron features after Fe introduction [28]. This result suggests a more electronically active local environment in the Fe‐regulated sample, which is favorable for interfacial charge redistribution and charge‐transfer processes (Figure 2l). This suggests that electron redistribution is more likely to occur near the interface. Together with the increased defect‐/hydroxyl‐related oxygen component in the O 1s spectrum, the enhanced defect‐sensitive signals suggest an increase in oxygen‐vacancy‐related sites after Fe introduction. This increase may be attributed to the modulation of the local Mn─O coordination and charge‐compensation environment by pre‐embedded Fe, which favors the formation or stabilization of oxygen‐deficient coordination configurations.

The kinetic behavior of the δ‐MnO_2_@Fe‐PCNFs electrode was evaluated by cyclic voltammetry (CV) at a scan rate of 0.1 mV s^−1^ within a potential window of 0.2–1.8 V (Figure 3a). The reduction peaks at ∼1.25 and ∼1.37 V and the oxidation peak at ∼1.55 V are associated with the reversible Zn^2+^/H^+^ insertion/extraction in layered δ‐MnO_2_, coupled with the Mn^4+^/Mn^3+^ redox transition. Notably, compared with δ‐MnO_2_@PCNFs, δ‐MnO_2_@Fe‐PCNFs exhibits similar redox peak positions (Figure 3b), indicating that Fe introduction does not fundamentally alter the electrode reaction pathway. However, the smaller peak shift observed for δ‐MnO_2_@Fe‐PCNFs suggests that Fe helps alleviate polarization fluctuations associated with the initial interfacial reconstruction, enabling the electrode to reach a stable working state more rapidly. This further confirms that Fe does not act as the primary redox‐active center. Consistent with the XRD results, Fe functions as an interfacial regulatory site rather than predominantly entering the lattice to modify the intrinsic redox chemistry of the electrode. As the scan rate increases, δ‐MnO_2_@Fe‐PCNFs exhibits a smaller peak shifts than δ‐MnO_2_@PCNFs, demonstrating superior charge‐transfer kinetics and ion‐response capability (Figure 3c and Figure S15). The charge storage kinetics were quantitatively analyzed using the power‐law relationship between peak current (i) and scan rate (v): i = a·v^b^, where the b value reflects the charge storage mechanism, with b = 0.5 corresponding to diffusion‐controlled behavior and b = 1.0 indicating surface‐controlled capacitive processes [33]. As shown in Figure 3d, the b values of δ‐MnO_2_@PCNFs are 0.53 and 0.51 for the oxidation and reduction peaks, respectively, slightly higher than those of pristine δ‑MnO_2_, suggesting a predominantly diffusion‐controlled process. In contrast, δ‐MnO_2_@Fe‐PCNFs exhibits significantly higher b values of 0.73 and 0.92, revealing that Fe regulation substantially reduces diffusion limitation and promotes a larger capacitive/interfacial contribution to Zn storage. The higher b‐values cannot be attributed solely to the PCNF substrate but are more likely associated with Fe‐induced improvements in the electrochemical accessibility of interfacial active sites and reducing the dependence on solid‐state diffusion (Figure S16). To further distinguish the capacitive and diffusion‐controlled contributions, the current response was separated according to: i = k_1_v + k_2_v^1/2^. At a scan rate of 0.1 mV s^−1^, the capacitive contribution accounts for 69.4% of the total charge storage in δ‐MnO_2_@Fe‐PCNFs, compared with only 53.1% in δ‐MnO_2_@PCNFs (Figure 3e) [56]. This pronounced increase indicates that Fe regulation significantly increases the availability of electrochemically active sites and promotes rapid surface Zn^2+^ storage, rather than relying primarily on sluggish solid‐state diffusion. As the scan rate increases from 0.1 to 0.5 mV s^−1^, the capacitive contribution progressively increases, suggesting that the Zn storage process becomes increasingly dominated by fast surface‐controlled reactions (Figure 3f). The higher capacitive contribution in δ‐MnO_2_@Fe‐PCNFs originates from Fe‐induced activation of redox‐active sites and facilitated interfacial charge transfer. Electrochemical impedance spectroscopy (EIS) and galvanostatic intermittent titration technique (GITT) were employed to further probe the reaction kinetics. The Nyquist plots consist of a semicircle in the high‐frequency region and an inclined line in the low‐frequency region, corresponding to the charge transfer resistance (R_ct_) and Warburg impedance associated with ion diffusion, respectively [57]. After 50 cycles at 1 A g^−1^, δ‐MnO_2_@Fe‐PCNFs exhibits a significantly lower R_ct_ (59.5 Ω) than δ‐MnO_2_@PCNFs (66.6 Ω) (Figure 3g and Table S4), indicating that Fe reduces the electrochemical charge‐transfer barrier by modulating the local Mn‐O environment and interfacial electronic structure, thereby improving reaction kinetic pathways and enabling faster Zn storage. The electronic conductivity of the electrodes was also evaluated. δ‐MnO_2_@Fe‐PCNFs exhibits a higher conductivity (3.77 × 10^−5^ S cm^−1^) compared to δ‐MnO_2_@PCNFs (2.69 × 10^−6^ S cm^−1^), indicating improved electron transport after Fe introduction. Consistently, GITT analysis reveals a higher Zn^2+^ diffusion coefficient for δ‐MnO_2_@Fe‐PCNFs (9.7 × 10^−13^ cm^2^ s^−1^) compared with δ‐MnO_2_@PCNFs (4.6 × 10^−13^ cm^2^ s^−1^) (Figure 3h and Table S5). This enhancement confirms that Fe‐mediated regulation of the local Mn─O environment, facilitates more efficient Zn^2+^ diffusion, and enables reversible insertion and extraction. Moreover, the wettability of the electrode toward the electrolyte was evaluated by contact angle measurements. The PCNFs exhibit a relatively large contact angle of 91.3°, indicating limited affinity toward the aqueous electrolyte. After δ‐MnO_2_ growth on the surface of PCNFs, the contact angle of δ‐MnO_2_@PCNFs significantly decreases, indicating enhanced surface polarity. Notably, δ‐MnO_2_@Fe‐PCNFs shows the smallest contact angle of 24.4°, indicating enhanced electrolyte affinity (Figure 3i). This improvement may be attributed to the increased surface polarity and defect‐rich environment induced by Fe regulation. The improved wettability facilitates electrolyte infiltration, thereby contributing to the accelerated Zn^2+^ reaction kinetics.

**FIGURE 3:**
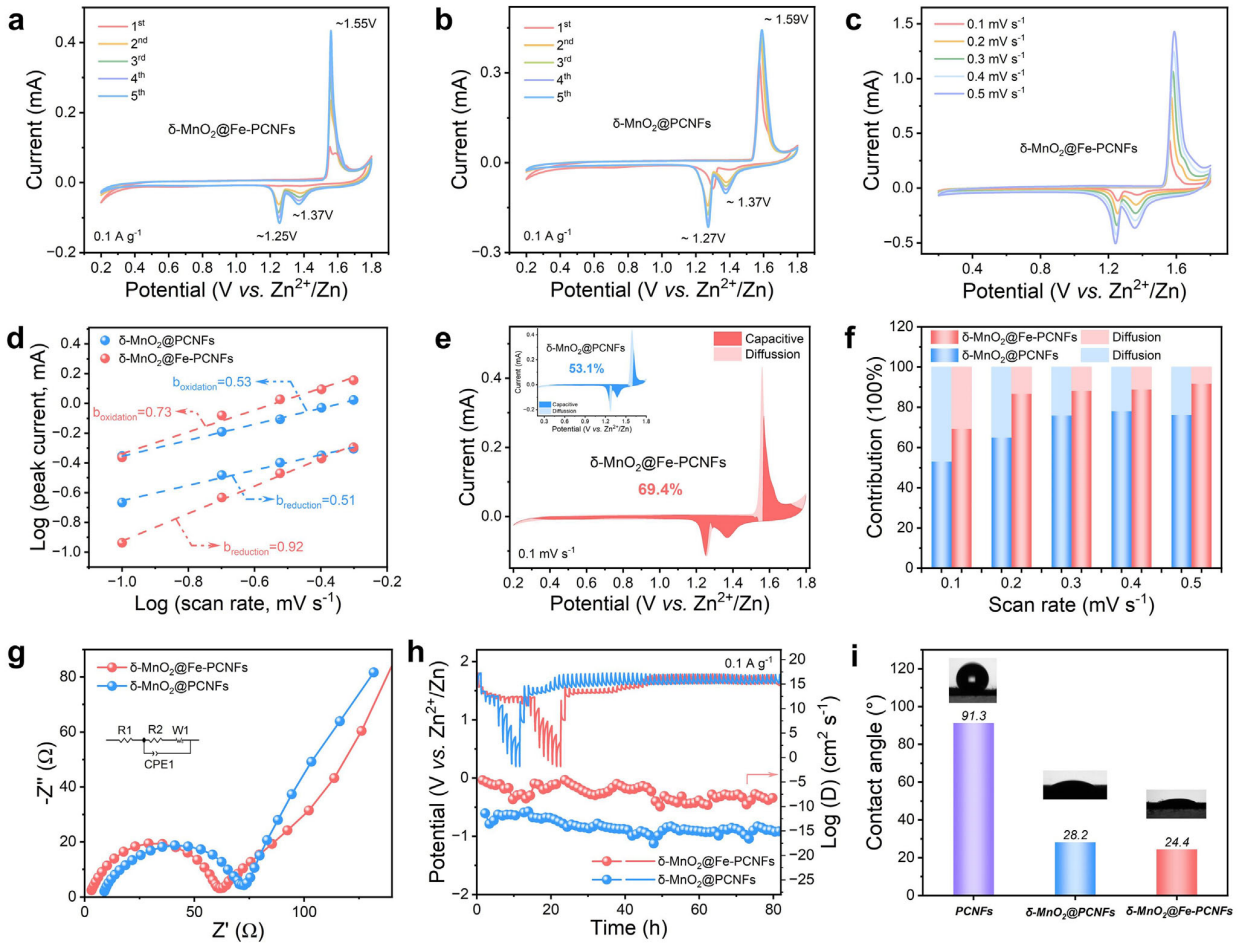
Interfacial accessibility and kinetic enhancement induced by Fe regulation. Initial five CV curves at a scan rate of 0.1 mV s^−1^ of (a) δ‐MnO_2_@Fe‐PCNFs and (b) δ‐MnO_2_@PCNFs. (c) CV curves at scan rates of 0.1–0.5 mV s^−1^ of δ‐MnO_2_@Fe‐PCNFs. (d) The b value of the oxidation and reduction peaks of the δ‐MnO_2_@Fe‐PCNFs and δ‐MnO_2_@PCNFs at different scan rates. The capacitive contribution of the δ‐MnO_2_@Fe‐PCNFs and δ‐MnO_2_@PCNFs (e) at 0.1 mV s^−1^ and (f) at different scan rates ranging from 0.1 to 0.5 mV s^−1^ of δ‐MnO_2_@Fe‐PCNFs and δ‐MnO_2_@PCNFs. (g) EIS curve at 1 A g^−1^ after 50 cycles and (h) GITT profiles at 0.1 A g^−1^ of δ‐MnO_2_@Fe‐PCNFs and δ‐MnO_2_@PCNFs. (i) The electrolyte contact angle of δ‐MnO_2_@Fe‐PCNFs, δ‐MnO_2_@PCNFs, and PCNFs.

To elucidate the reversible evolution of the δ‐MnO_2_@Fe‐PCNFs electrode in AZIBs, various ex situ characterizations were conducted to track crystal structure evolution and elemental valence state changes at different charge/discharge states. Figure 4a,b illustrate the ex situ XRD patterns of the δ‐MnO_2_@Fe‐PCNFs and δ‐MnO_2_@PCNFs electrodes at different stages during the charge/discharge process. During discharging, a new diffraction peak emerges at ∼9.5°, corresponding to the formation of zinc hydroxysulfate (ZHS; Zn_4_SO_4_(OH)_6_·xH_2_O) [58]. The intensity of this peak gradually increases from 1.3 to 0.2 V and diminishes during the subsequent charging process. Upon charging to 1.7 V, the ZHS signal disappears completely, indicating excellent reversibility of this intermediate phase. In contrast, δ‐MnO_2_@PCNFs without Fe modulation shows a pronounced increase in ZHS peak intensity during discharge, accompanied by significant peak broadening. Even after charging to 1.8 V, residual ZHS signals remain, suggesting irreversible structural degradation. This further demonstrates that Fe effectively maintains the reversible evolution of the δ‐MnO_2_ framework, enhances the mechanical stability of the layered structure, alleviates the accumulation of parasitic reactions, and suppresses the nucleation and growth of ZHS. Ex situ Raman spectroscopy provides direct evidence for reversible structural evolution (Figure 4c). During discharge, Zn^2+^/H^+^ insertion induces partial reduction of Mn^4+^ to Mn^3+^. The associated Jahn‐Teller distortion weakens the Mn─O bond at ∼645 cm^−1^, decreasing its intensity and shifting the Raman band to a lower wavenumber [59]. During the charging process, Zn^2+^/H^+^ extraction restores Mn^3+^ to Mn^4+^, and the Mn─O vibrational peak recovers its original position and intensity. This directly demonstrates that the local Mn─O framework in δ‐MnO_2_@Fe‐PCNFs undergoes highly reversible reconstruction under Fe‐mediated surface regulation. To further monitor Zn^2+^ insertion/extraction, ex situ XPS measurements were performed at fully discharged (0.2 V) and fully charged (1.8 V) states. The high‐resolution Zn 2p spectra (Figure 4d) show two characteristic peaks at 1023.8 eV (Zn 2p_3/2_) and 1046.8 eV (Zn 2p_1/2_) after discharge, confirming successful Zn^2+^ insertion into the electrode. Upon charging to 1.8 V, the Zn 2p signal intensity decreases markedly and shifts toward lower binding energy, indicating reversible Zn^2+^ extraction, while a small fraction remains due to strong Zn─O interactions. The ex situ Mn 2p spectra further reveal the redox behavior of Mn (Figure 4e). After discharge to 0.2 V, the Mn 2p peaks shift toward lower binding energy by ∼0.6 eV, accompanied by an increased Mn^3+^ contribution, indicating reduction of Mn^4+^ during Zn^2+^ insertion [29]. Upon charging, the Mn 2p peaks return to their initial positions, demonstrating reversible oxidation of Mn^3+^ to Mn^4+^. This supports the involvement of a highly reversible Mn^4+^/Mn^3+^ redox couple in the Zn storage. The O 1s spectra provide further insight into the reaction mechanism. During discharging, the relative contribution of lattice oxygen slightly decreases, while the proportion of defect‐related oxygen and adsorbed hydroxyl/water species increases, indicating the involvement of H^+^/H_2_O in the Zn storage process (Figure 4f). After charging, the lattice oxygen signal is restored, demonstrating the reversibility of the local oxygen environment. Overall, the state‐resolved XRD, Raman, and XPS results show that Fe regulation does not fundamentally alter the Mn‐based redox chemistry, but promotes a more reversible structural and chemical response during charge and discharge. The correlated recovery of the phase composition, local Mn─O environment, Mn valence state, and surface Zn/O chemistry, together with the more complete disappearance of the ZHS‐related diffraction signal upon charging, indicates reduced accumulation of irreversible reaction products and improved structural reversibility in δ‐MnO_2_@Fe‐PCNFs. In addition, ICP‐OES analysis was conducted to quantify Mn dissolution during cycling. After 100 cycles, the retained Mn content in δ‐MnO_2_@Fe‐PCNFs (8.82%) is significantly higher than that in δ‐MnO_2_@PCNFs (4.95%) (Figure 4g). Correspondingly, the Mn concentration in the electrolyte of the δ‐MnO_2_@Fe‐PCNFs remains consistently lower and decreases with prolonged cycling, whereas δ‐MnO_2_@PCNFs shows an increasing trend. These complementary results demonstrate that Fe regulation effectively mitigates Mn loss and improves Mn retention during cycling, thereby contributing to stabilization of the Mn─O framework and preservation of structural integrity during repeated Zn^2+^ insertion/extraction processes. Therefore, the improved electrochemical durability is associated with a more stable interfacial reaction environment, rather than merely enhanced initial activity. Correspondingly, the δ‐MnO_2_@Fe‐PCNFs electrode still maintains an intact fibrous structure after 200 cycles, as observed by SEM and TEM (Figure 4h,i). The influence of Fe regulation on stability was further evaluated by examining the morphological evolution of the Zn anode after 10, 50, and 100 cycles (Figure 4j). With increasing cycling, the Zn surface in the δ‐MnO_2_@PCNFs becomes progressively rough, eventually developing into abundant and uneven dendritic deposits, indicating an increasingly unbalanced Zn plating/stripping process. In contrast, the Zn anode paired with δ‐MnO_2_@Fe‐PCNFs exhibits a significantly smoother and denser surface morphology, with dendrite growth effectively suppressed even after prolonged cycling. This behavior can be attributed to the Fe‐regulated suppression of Mn dissolution and reduced cell polarization, which together provide a more stable deposition environment for Zn and enable uniform Zn plating/stripping behavior. Therefore, Fe does not alter the intrinsic Zn storage pathway of δ‐MnO_2_, but enhances its reversibility in terms of structural evolution, bonding environment, and chemical‐state variation. This improved reversibility suppresses continuous Mn loss, preserves cathode integrity, and stabilizes the electrode/electrolyte interface during cycling.

**FIGURE 4:**
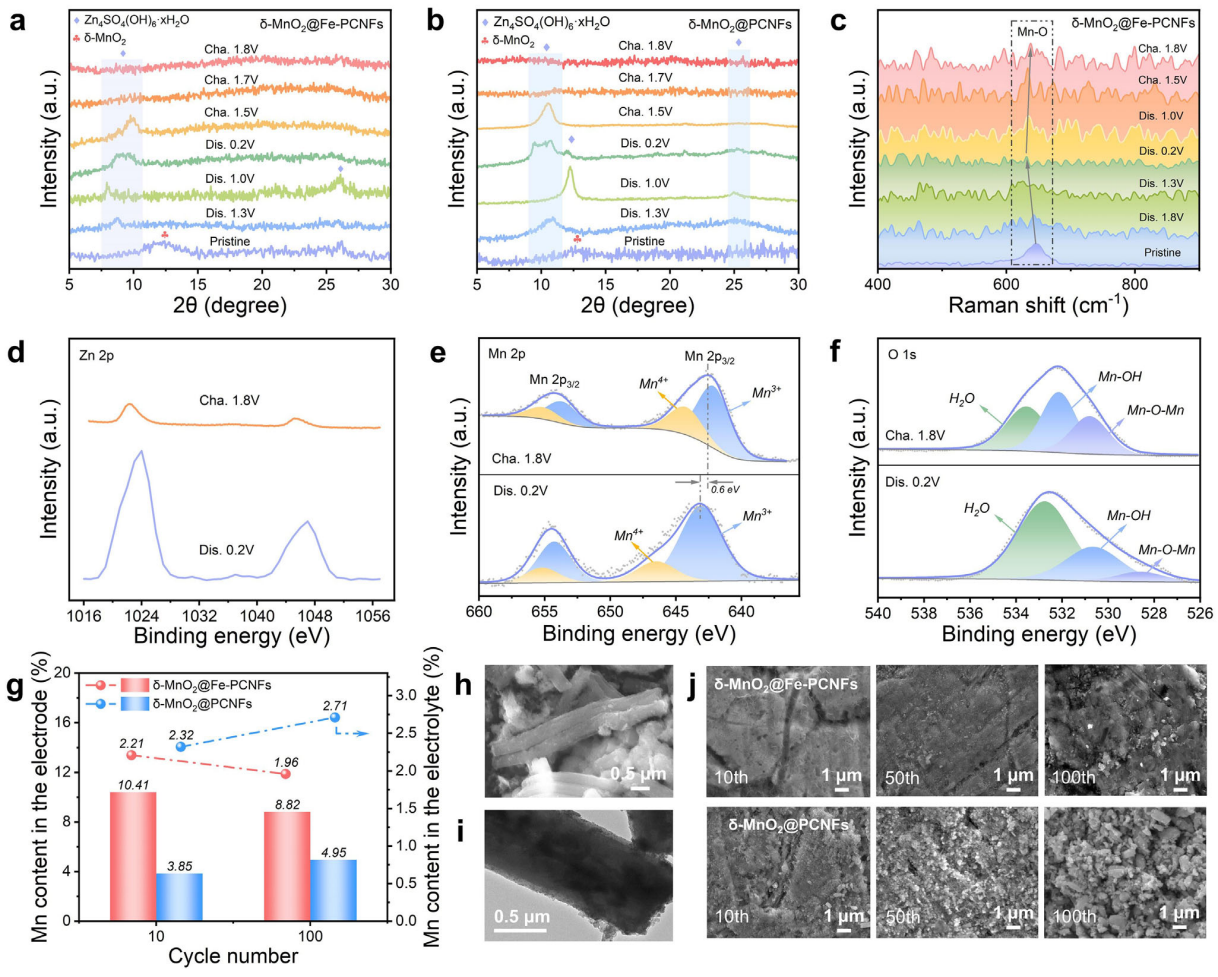
Cycling‐induced structural evolution and stability analysis. (a) Ex situ XRD patterns at various charge/discharge processes of (a) δ‐MnO_2_@Fe‐PCNFs and (b) δ‐MnO_2_@PCNFs. (c) Ex situ Raman spectra of δ‐MnO_2_@Fe‐PCNFs at various discharging/charging processes. High‐resolution (d) Zn 2p, (e) Mn 2p, and (f) O1s XPS spectra of δ‐MnO_2_@Fe‐PCNFs after charging to 1.8 V and discharging to 0.2 V. (g) The Mn content in the electrode and electrolyte of δ‐MnO_2_@Fe‐PCNFs and δ‐MnO_2_@PCNFs after the 10th and 100th cycles. (h) SEM and (i) TEM images of δ‐MnO_2_@Fe‐PCNFs after 200 cycles. (j) SEM images of zinc sheets after 10, 50, and 100 cycles of δ‐MnO_2_@Fe‐PCNFs and δ‐MnO_2_@PCNFs.

The initial galvanostatic charge–discharge (GCD) profiles of δ‐MnO_2_@Fe‐PCNFs and δ‐MnO_2_@PCNFs exhibit nearly identical voltage plateaus, indicating that the introduction of Fe does not alter the intrinsic Zn storage mechanism. Both electrodes deliver an initial Coulombic efficiency of ∼ 85.5%, indicating reversible Zn^2+^ storage behavior (Figure 5a). The initial Coulombic inefficiency likely arises from electrode activation and interfacial evolution, with possible contributions from parasitic reactions inherent to aqueous Zn chemistry, such as hydrogen evolution and Zn corrosion. The subsequent evolution of the Coulombic efficiency suggests progressively stabilized and more reversible electrochemical behavior. The rate capability of the δ‐MnO_2_@Fe‐PCNFs electrode was evaluated at current densities ranging from 0.2 to 10 A g^−1^ (Figure 5b). The δ‐MnO_2_@Fe‐PCNFs electrode exhibits a strong rate response, delivering an initial discharge capacity of 409.29 mAh g^−1^ at 0.2 A g^−1^ and average capacities of 267.16, 203.00, 154.74, and 99.12 mAh g^−1^ at 0.5, 1, 2, and 5 A g^−1^, respectively, significantly outperforming δ‐MnO_2_@PCNFs, Mn, Fe@PCNFs, Fe‐PCNFs, and pristine δ‐MnO_2_ (Figures S17 and S18). Even at a high current density of 10 A g^−1^, δ‐MnO_2_@Fe‐PCNFs still retains a specific discharge capacity of 52.17 mAh g^−1^. In addition, the differential capacity profiles collected at 0.2 A g^−1^ consistently show that the Fe‐regulated δ‐MnO_2_@Fe‐PCNFs electrode delivers a significantly larger reversible redox contribution (Figure 5c). In contrast, the δ‐MnO_2_@PCNFs electrode exhibits a larger separation between the corresponding cathodic and anodic features, indicating more pronounced electrochemical polarization. Therefore, the capacity enhancement of δ‐MnO_2_@Fe‐PCNFs can be attributed to the broader and more reversible Mn‐centered redox reactions enabled by Fe interfacial regulation. At higher current densities, the reduced electrochemical polarization and improved charge‐transfer and ion‐transport characteristics become increasingly important, which accounts for the superior rate capability of the Fe‐regulated electrode. At a current density of 1 A g^−1^, the δ‐MnO_2_@Fe‐PCNFs electrode maintains a specific capacity of 243.64 mAh g^−1^ after 1000 cycles (Figure 5d). The gradual capacity increase observed during prolonged cycling is also associated with Mn^2+^‐dependent dissolution‐redeposition chemistry in the MnSO_4_‐containing electrolyte, which can provide an additional reversible redox contribution to the measured capacity (Figure S19) [60, 61, 62]. Even at a high current density of 5 A g^−1^, it maintains a capacity retention of 81.1% after 3000 cycles (Figure S20). This stability arises from Fe modulation, which effectively stabilizes the Mn─O framework, while the carbon nanofiber matrix provides robust mechanical support to buffer volume variations of δ‐MnO_2_ during repeated Zn^2+^ insertion/extraction. In contrast, pristine δ‐MnO_2_, without the structural support of carbon nanofibers and interfacial regulation by Fe, exhibits rapid capacity fading during cycling. Compared with pristine δ‐MnO_2_, δ‐MnO_2_@PCNFs maintains substantially higher capacities over the investigated current‐density range, with the advantage becoming more pronounced at elevated rates. This behavior highlights the role of the interconnected PCNFs framework in maintaining electrode‐level reaction accessibility and active‐material utilization under increasing current demand. Compared with previously reported MnO_2_‐based AZIBs cathodes derived from conventional bulk doping or structural engineering strategies, δ‐MnO_2_@Fe‐PCNFs achieve enhanced specific capacity through interfacial electronic regulation (Figure 5e and Table S6) [13, 52, 58, 63, 64, 65, 66, 67, 68, 69, 70]. Figure 5f illustrates the role of Fe in regulating Zn storage behavior. In the absence of pre‐embedded Fe, δ‐MnO_2_@PCNFs exhibits disordered growth of δ‐MnO_2_ nanosheets, limited interfacial adhesion, and irregular diffusion pathways, resulting in sluggish Zn^2+^ diffusion. This is accompanied by structural degradation of the δ‐MnO_2_ nanosheets and severe Mn dissolution. In contrast, pre‐embedded Fe regulates the nucleation and growth interface of δ‐MnO_2_, promoting the formation of a more integrated nanosheet architecture, a more accessible local Mn─O environment, and stronger interfacial coupling between δ‐MnO_2_ and the carbon nanofiber framework in δ‐MnO_2_@Fe‐PCNFs. The optimized interface accelerates coupled electron and ion transport while providing accessible interlayer Zn^2+^ diffusion pathways, thereby enabling efficient and reversible Zn storage. These advantages collectively enhance reaction kinetics and structural stability, contributing to the observed rate capability and stable cycling performance. Additionally, the δ‐MnO_2_@Fe‐PCNFs membrane exhibits certain mechanical flexibility, maintaining structural integrity under bending, folding, and winding conditions (Figure S21), highlighting its potential for flexible energy storage applications. To further evaluate the electrochemical behavior under increased electrode loading, a δ‐MnO_2_@Fe‐PCNFs electrode with a mass loading of approximately 3.1 mg cm^−2^ was investigated (Figure S22). Although the increased electrode thickness intensifies mass‐transport limitations, the high‐loading electrode maintained nearly 100% capacity retention after 1000 cycles at a current density of 1 A g^−1^. To further evaluate the performance of δ‐MnO_2_@Fe‐PCNFs in a larger‐area device configuration, a pouch cell was assembled using a 4 cm^2^ cathode containing approximately 5 mg of composite material (Figure 5g and Figure S23). The pouch cell maintained reversible charge/discharge responses over different current densities and showed good capacity recovery when the current density was subsequently reduced. Moreover, the device was capable of continuously powering a light‐emitting diode, demonstrating that δ‐MnO_2_@Fe‐PCNFs can be extended from conventional coin‐cell testing to a larger‐area pouch‐cell configuration. Together with the high‐loading electrode results, these observations further demonstrate the feasibility of implementing the Fe‐regulated electrode architecture under increased electrode area and active‐material loading.

**FIGURE 5:**
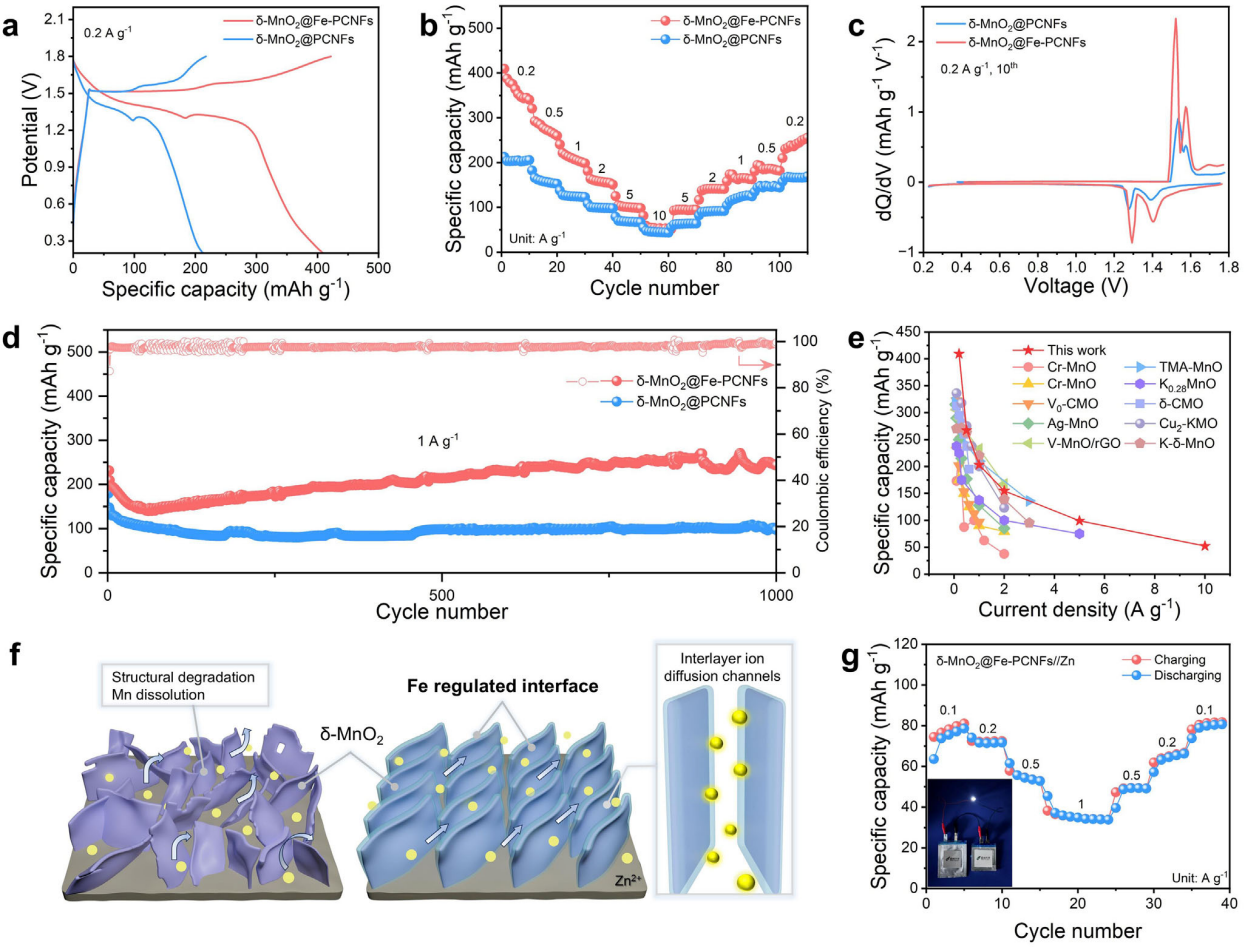
Electrochemical performance and device‐level evaluation of δ‐MnO_2_@Fe‐PCNFs. (a) Initial discharging and charging profiles of δ‐MnO_2_@Fe‐PCNFs and δ‐MnO_2_@PCNFs at 0.2 A g^−1^. (b) Rate capacity at different current densities, (c) differential capacity (dQ/dV) profiles at 0.2 A g^−1^ during the 10th cycle, and (d) long cycling performance at 1 A g^−1^ of δ‐MnO_2_@Fe‐PCNFs and δ‐MnO_2_@PCNFs. (e) A comparison of the rate performance with that of the previously reported MnO_2_‐based cathode in AZIBs. (f) Schematic illustration of the synergistic mechanism of δ‐MnO_2_@Fe‐PCNFs and δ‐MnO_2_@PCNFs. (g) Rate performance of pouch batteries with δ‐MnO_2_@Fe‐PCNFs.

## 3 Conclusion

In summary, we demonstrate a growth‐interface regulation strategy for δ‐MnO_2_ cathodes in aqueous Zn‐ion batteries. Rather than reconstructing the bulk lattice, a small amount of Fe intervenes in the formation process of δ‐MnO_2_, modulating its nucleation and growth behavior and the local Mn─O environment. Combined theoretical and experimental results indicate that Fe is predominantly present as highly dispersed, oxidized surface‐/interface‐associated species. Rather than fundamentally altering the Mn^4+^ /Mn^3+^‐dominated redox chemistry, Fe regulates the local Mn─O environment and interfacial reaction characteristics, thereby improving Zn‐storage accessibility, structural reversibility, and cycling stability. This interfacial regulation simultaneously accelerates Zn^2+^ storage kinetics and suppresses Mn dissolution, contributing to an initial discharge capacity of 409.29 mAh g^−1^ at 0.2 A g^−1^ and a capacity of 243.64 mAh g^−1^ at 1 A g^−1^ after 1000 cycles, maintaining a capacity retention of 81.1% after 3000 cycles at 5 A g^−1^. This work highlights growth‐interface regulation as an effective route to simultaneously improve reaction kinetics and structural durability in Mn‐based aqueous Zn‐ion battery cathodes.

## 4 Experimental Section

### 4.1 Preparation of δ‐MnO_2_@Fe‐PCNFs

1 g polyacrylonitrile and 0.4 g polymethyl methacrylate was completely dissolved in 8 mL N, N‐dimethylformamide (DMF) with vigorous stirring at 60°C to obtain a homogeneous solution A. 1 g of MnCl_2_·4H_2_O, 0.2 g of FeCl_3_·6H_2_O, and 0.5 g of Acetylacetone solution were added to 4 mL of DMF solution and heated at 60°C with stirring to obtain a homogeneous solution B. Solution B was poured into solution A and stirred uniformly to obtain the spinning solution. The spinning solution was loaded into a syringe and electrospun using an appropriate needle, a suitable flow rate of 0.5 mL h^−1^, and an applied voltage of 15 kV, yielding a uniform electrospun membrane after several hours. The electrospun membrane was heated to 250°C in air and held for 2 h for pre‐oxidation, resulting in a stabilized pre‐oxidized membrane. The pre‐oxidized membrane was then heated to 300°C and held for 1 h, then heated to 600°C and held for 2 h under an Ar atmosphere for carbonization, obtaining a carbonized membrane (Mn, Fe@PCNFs). The carbonized membrane was immersed in a solution containing 0.05 M KMnO_4_ and 0.05 M ammonium persulfate for 2 h. Subsequently, the sample was rinsed with deionized water to remove residual solution and dried at 80°C for 2 h, and then heated to 150°C for 1 h to stabilize the crystal structure, yielding δ‐MnO_2_@Fe‐PCNFs. Without adding FeCl_3_·6H_2_O to the spinning solution, δ‐MnO_2_@PCNFs were prepared as a control sample following the same procedure. Using only solution A as the spinning solution, porous carbon nanofibers were obtained through the same pre‐oxidation and carbonization processes. Furthermore, without adding MnCl_2_·4H_2_O to spinning solution B, Fe‐PCNFs control samples were obtained after the pre‐oxidation and carbonization processes.

An aqueous solution of KMnO_4_ (0.05 M) was added dropwise to 150 mL of an alkaline aqueous solution containing MnCl_2_·4H_2_O (0.05 M) under vigorous stirring at room temperature. The reaction mixture was continuously stirred for 24 h to ensure complete precipitation. The resulting precipitate was collected by centrifugation, thoroughly washed with deionized water until a neutral pH was reached, and then dried at low temperature to obtain pure δ‐MnO_2_.

### 4.2 Characterization

The morphology and microstructure were observed by field‐emission scanning electron microscopy (FE‐SEM, JSM‐IT800SHL, JEOL) and transmission electron microscopy (JEM‐ARM2010F, JEOL). The band gap of the sample was measured using a UV–vis reflectance spectrophotometer (V‐750, JASCO) with the wavelength range of 300–550 nm. X‐ray diffraction (MiniFlex300, Rigaku Corporation) with Cu‐Kα radiation between 5° and 90° was used to investigate the crystal structure of the samples. The chemical valence state and composition of the composites were investigated by X‐ray photoelectron spectroscopy (PHI GENESIS, ULVAC‐PHI, Inc.). To verify the introduction of vacancies, electron paramagnetic resonance spectroscopy (Bruker, A300 EMX PLUS) was used to detect oxygen vacancies in the sample. Raman spectra were using a Hololab 5000 spectrometer with a laser excitation wavelength of 532 nm to analyze the molecular structure and chemical bond composition of the sample. The pore size and specific surface area of the composites were calculated by BET analysis (TriStar II, Micromeritics). The Mn and Fe content were determined using an inductively coupled plasma emission spectrometer (SPS3100, SII NanoTechnology Inc.).

### 4.3 Electrochemical Measurements

A slurry was prepared by mixing the active material, conductive carbon black, and poly(vinylidene fluoride (PVDF) at a mass ratio of 7:2:1. The resulting homogeneous slurry was uniformly coated onto Ti foil as the current collector and subsequently dried under vacuum at 100°C for 12 h. The active‐material loading was approximately 0.7 mg cm^−2^, and the specific capacity was calculated based on the mass of the composite active material, excluding conductive carbon black and PVDF. Glass fiber (Whatman GF/D) was used as the separator, zinc foil as the anode, and an aqueous solution containing 2 M ZnSO_4_ and 0.5 M MnSO_4_·H_2_O as the electrolyte. CR2032 coin cells were assembled using 180 µL of electrolyte per cell for electrochemical measurements. CV and EIS measurements were performed on an electrochemical workstation (Biologic) within a voltage range of 0.2–1.8 V and a frequency range of 0.01–100 kHz. GCD tests were conducted on a battery testing system (LAND, China).

## Author Contributions

**Fanghua Liang**: conceptualization, writing – original draft, investigation, validation, methodology, visualization, data curation. **Ling Wang**: formal analysis, investigation. **Junpeng Xiong**: formal analysis, validation. **Chunqing Niu**: validation, investigation. **Atsuya Kawai**: investigation. **Wei Zhang**: supervision, resources. **Chunhong Zhu**: resources. **Azeem Ullah**: resources, supervision. **Mayakrishnan Gopiraman**: writing – review and editing, resources, project administration, supervision. **Mingzheng Ge**: writing – review and editing, resources, supervision. **Ick Soo Kim**: conceptualization, writing – review and editing, resources, supervision, funding acquisition, project administration.

## Conflicts of Interest

The authors declare no conflicts of interest.

## Supporting information




**Supporting File**: advs78465‐sup‐0001‐SuppMat.docx.

## Data Availability

Data will be made available on request.
