# Supplementary material for: Formation‐Stage Interfacial Regulation of δ‐MnO2 Enables Stable and Reversible Aqueous Zn‐Ion Storage
Source: Adv Sci (Weinh). 2026 Nov 4:e78465. Online ahead of print. doi: 10.1002/advs.78465 (PMC13736147; doi:10.1002/advs.78465)
Supplement: Supplementary file 1 [file ADVS-9999-e78465-s001.docx]

Supporting Information

**Formation-Stage Interfacial Regulation of δ-MnO_2_ Enables Stable and Reversible Aqueous Zn-Ion Storage**

*Fanghua Liang^a^, Ling Wang^a^, Junpeng Xiong^a^, Chunqing Niu^a^, Atsuya Kawai^a^, Wei Zhang^b^, Chunhong Zhu^a^, Azeem Ullah^a^, Mayakrishnan Gopiraman^a,^*, Mingzheng Ge^b,^*, Ick Soo Kim^a,^**

F. Liang, L. Wang, J. Xiong, C. Niu, A. Kawai, Prof. C. Zhu, A. Ullah, Prof. M. Gopiraman, Prof. I. Kim

^a^Nano Fusion Technology Research Lab, Institute for Fiber Engineering and Science (IFES), Research Cluster for Social Implementation, Shinshu University, Tokida 3-15-1, Ueda, Nagano 386-8567, Japan

Prof. W. Zhang, Prof. M. Ge

^b^School of Textile and Clothing, Nantong University, Nantong 226019, P. R. China

*Corresponding authors. E-mail address: kim@shinshu-u.ac.jp (I.S. Kim); mzge1990@ntu.edu.cn (M. Ge); gopiraman@shinshu-u.ac.jp (M. Gopiraman);

**1. Supporting Notes**

**1.1. Band gap measurement by the diffuse reflectance spectrum (UV-Vis DRS)**

Band gap measurement typically requires determining the diffuse reflectance spectrum (UV-Vis DRS) of a solid sample, and then calculating the band gap value using the Tauc plot method. The measured reflectance data is converted into an absorption spectrum using the Kubelka-Munk function [1]:

$$F(R)=\frac{\left(1-R\right)^{2}}{2R}=\frac{\alpha}{\text{s}}$$

where R is the reflectance, α is the absorption coefficient, and s is the scattering coefficient. The Tauc plot is obtained by plotting the relationship between (F(R)⋅ h*v*)^2^ and photon energy h*v*, and extrapolating the linear portion of the curve to the horizontal (photon-energy) axis gives the optical band gap energy E_g_. The photon energy is calculated as h*v* =1240/λ, where λ is the wavelength (nm).

**1.2. Zinc ionic diffusion coefficient estimated by galvanostatic intermittent titration technique (GITT)**

To further study the zinc ion diffusion properties ($D_{Z\text{n}^{2+}}$) of δ-MnO_2_@Fe-PCNFs, it was calculated by GITT using the equation as follows [2]:

$$D_{Z\text{n}^{2+}}=\frac{4}{\pi t}\left(\frac{m_{B}V_{m}}{M_{B}S}\right)^{2}\left(\frac{\Delta E_{S}}{\Delta E_{\text{t}}}\right)^{2}$$

Where *t* is the relaxation time, $V_{m}$ is the molar volume, $m_{B}$ is the mass loading of electrode, *S* is the electrode area, *M_B_* is the molar mass, $\Delta E_{S}$ is the voltage change, and $\Delta E_{t}$ is the voltage change during constant-current charge and discharge. In this GITT test, the battery was charged/discharged at 0.1 A g^-1^ for 600 s, and then relaxed under open circuit for 1 h.

**1.3. Theoretical analysis of Zn-ion adsorption behavior in the electrode.**

First-principles calculations were performed using the CASTEP module in Materials Studio based on density functional theory (DFT) to investigate the ion adsorption behavior and underlying interaction mechanism on the δ-MnO_2_ surface [3]. The computational model was first constructed based on the correct layered δ-MnO_2_ structure. An oxygen-terminated monolayer surface slab was cleaved along the (001) direction to describe the local adsorption behavior and electronic structure of the exposed δ-MnO_2_ (001) surface. A 15 Å vacuum layer was introduced along the surface-normal direction to eliminate interactions between periodic images. All atoms were fully relaxed during structural optimization. The electron exchange–correlation interactions were described using the Perdew–Burke–Ernzerhof (PBE) functional within the generalized gradient approximation (GGA) [4]. The interactions between ionic cores and valence electrons were treated using ultrasoft pseudopotentials, and spin-polarized calculations were performed [5]. After convergence tests, the plane-wave cutoff energy was set to 450 eV, and the Brillouin zone was sampled using a Monkhorst–Pack k-point mesh of 3 × 3 × 1. The convergence criteria for structural optimization were set as follows: energy convergence threshold ≤ 1.0 × 10^-5^ eV atom^-1^, force convergence threshold ≤ 0.03 eV Å^-1^, and atomic displacement convergence threshold ≤ 0.001 Å. Based on the optimized stable surface models, ion adsorption energies, density of states, and band structures were calculated.

**2. Supporting Figures**

**Figure S1** DFT structural models of the structural model of a) δ-MnO_2_, b) δ-MnO_2_@Fe, and c) Fe_Mn_-δ-MnO_2_.

**Figure S2** Band structures of a) δ-MnO_2_, b) Fe_Mn_-δ-MnO_2_, and c) δ-MnO_2_@Fe.

**Figure S3** a) UV-vis DRS and b) Tauc plots of δ-MnO_2_@Fe-PCNFs and δ-MnO_2_@PCNFs.

**Figure S4** The charge density difference plot of a) δ-MnO_2_, b) Fe_Mn_-δ-MnO_2_, and c) δ-MnO_2_@Fe.

**Figure S5** SEM images of δ-MnO_2_@Fe-PCNFs and δ-MnO_2_@PCNFs soaked in KMnO_4_ solution for 10, 30, and 60 min to show the time-dependent immersion experiments.

**Figure S6** a) SEM, b) TEM, c) SAED, and d) EDX images of δ-MnO_2_@PCNFs.

**Discussion**: The δ-MnO_2_@PCNFs composite exhibits an average fiber diameter of ~ 420 nm, with δ-MnO_2_ nanosheets growing in a disordered manner on the surface. EDX mapping confirms the uniform distribution of C, N, O, and Mn elements throughout the composite.

**Figure S7** N_2_ adsorption-desorption isotherms and pore size distributions of PCNFs, δ-MnO_2_@PCNFs, and δ-MnO_2_@Fe-PCNFs.

**Figure S8** SAED of the δ-MnO_2_@Fe-PCNFs.

**Figure S9** STEM-EDX of the δ-MnO_2_@Fe-PCNFs.

**Figure S10** a) STEM-HAADF image of the δ-MnO_2_@Fe-PCNFs and the line shows the area from which EELS line profiles have been collected. b) EELS spectra and c) Mn-L_3_/L_2_ and Fe-L_3_/L_2_ edge ELNES spectra of δ-MnO_2_@Fe-PCNFs.

**Figure S11** SEM images of a) δ-MnO_2_@Fe-PCNFs (0.05Fe), b) δ-MnO_2_@Fe-PCNFs (0.2Fe), c) δ-MnO_2_@Fe-PCNFs (0.4Fe), d) δ-MnO_2_@Fe-PCNFs (0.6Fe).

**Discussion**: To systematically evaluate the Fe-content dependence, we prepared a series of samples using 0, 0.05, 0.20, 0.40, and 0.60 g FeCl_3_·6H_2_O during precursor preparation. It should be noted that the amount of Fe salt added to the precursor does not directly correspond to the actual Fe mass fraction retained in the final composite. Because quantitative ICP results for the final Fe contents of all gradient samples are currently unavailable, we labeled these samples according to the actual mass of FeCl_3_·6H_2_O added, such as δ-MnO_2_@Fe-PCNFs (0.05Fe). SEM observations show that moderate Fe introduction favors a more uniform δ-MnO_2_-covered fibrous architecture, whereas excessive Fe increasingly disrupts the balanced nucleation/growth process and produces less uniform surface coverage.

**Figure S12** a) UV-vis DRS and b) Tauc plots of δ-MnO_2_@Fe-PCNFs with different Fe additions (0.05, 0.4 and 0.6g). c) Rate capacity at different current densities, and d) Long cycling performance of δ-MnO_2_@Fe-PCNFs with different Fe additions (0.05, 0.2, 0.4 and 0.6g).

**Discussion**: Consistently, the optical gap decreases from 2.23 eV for the Fe-free sample to 1.81 eV at 0.05 g and reaches a minimum of 1.68 eV at the optimized 0.20 g condition. It remains low at 0.40 g (1.71 eV) but increases markedly to 2.41 eV at 0.60 g, demonstrating that the electronic modulation is not proportional to Fe precursor addition. The electrochemical results show an even clearer optimum. The 0.20 g Fe sample exhibits the highest capacity over the entire tested current-density range, the strongest capacity recovery after returning to low current density, and the highest capacity during prolonged cycling at 1 A g^-1^. Lower Fe addition (0.05 g) provides only partial improvement, whereas further increasing the Fe precursor to 0.40 and 0.60 g progressively deteriorates both rate capability and cycling performance. Notably, the similar optical gaps of the 0.20 and 0.40 g samples but their substantially different electrochemical performance indicate that band-gap narrowing alone does not determine the Zn-storage behavior; an appropriate Fe-regulated growth/interface configuration is also required. Taken together, the gradient study establishes 0.20 g FeCl_3_·6H_2_O as the optimal precursor-feed condition, at which electronic/defect regulation and δ-MnO_2_ growth are most effectively balanced.

**Figure S13** Quantitative analysis of the XRD features of δ-MnO_2_@Fe-PCNFs and δ-MnO_2_@PCNFs.

**Figure S14** XPS of δ-MnO_2_@Fe-PCNFs, δ-MnO_2_@PCNFs, Mn, Fe@PCNFs, and PCNFs. High-resolution b) Mn 2p and c) Fe 2p XPS spectra of δ-MnO_2_@Fe-PCNFs.

**Figure S15** The CV curves at scan rates of 0.1-0.5 mV s^-1^ of δ-MnO_2_@PCNFs.

**Figure S16** a) CV curves at scan rates of 0.1-0.5 mV s^-1^ of δ-MnO_2_. b) The b value of the oxidation and reduction peaks of δ-MnO_2_, δ-MnO_2_@Fe-PCNFs and δ-MnO_2_@PCNFs at different scan rates. c) The capacitive contributions of δ-MnO_2_, δ-MnO_2_@Fe-PCNFs and δ-MnO_2_@PCNFs at different scan rates ranging from 0.1 to 0.5 mV s^-1^.

**Figure S17** SEM images of a) Mn, Fe@PCNFs, b) Fe-PCNFs, c) PCNFs, and d) δ-MnO_2_.

**Figure S18** a) The rate capacity at different current densities and b) long cycling performance of Mn, Fe@PCNFs, Fe-PCNFs, PCNFs, and δ-MnO_2_.

**Figure S19** a) The rate capacity at different current densities and b) long cycling performance of δ-MnO_2_@ Fe-PCNFs in 2 M ZnSO_4_ + 0.5 M MnSO_4_ and in Mn^2+^-free 2 M ZnSO_4_ electrolyte.

**Discussion**: Additional control tests were performed using the same δ-MnO_2_@Fe-PCNFs electrode in an electrolyte containing only 2 M ZnSO_4_ without Mn^2+^. The electrode still exhibited a reversible rate response in the Mn^2+^-free electrolyte, and the capacity recovered when the current density was reduced. This result indicates that δ-MnO_2_@Fe-PCNFs maintains electrochemical activity even without externally supplied Mn^2+^, confirming that the measured capacity does not originate entirely from Mn species provided by the electrolyte. In the MnSO_4_-containing electrolyte, δ-MnO_2_@Fe-PCNFs showed an initial capacity decrease followed by gradual capacity recovery during long-term cycling at 1 A g^-1^. In contrast, the electrode cycled in the Mn^2+^-free 2 M ZnSO_4_ electrolyte exhibited a much lower but relatively stable capacity, and the pronounced capacity increase was largely absent. This comparison provides strong evidence that Mn^2+^-dependent contributions play a significant role in both the measured total capacity and the gradual capacity increase observed during long-term cycling.

**Figure S20** Long cycling performance of δ-MnO_2_@Fe-PCNFs and δ-MnO_2_@PCNFs at 5 A g^-1^.

**Discussion**: δ-MnO_2_@Fe-PCNFs delivers a discharge capacity of 59.58 mAh g^-1^ after 3000 cycles at 5 A g^-1^. For the long-term cycling analysis, the capacity retention was calculated relative to the first cycle included in the sustained 5 A g^-1^ cycling segment, which delivered 73.47 mAh g^-1^. The immediate transition response upon switching to 5 A g^-1^ was not used as the retention reference. Accordingly, δ-MnO_2_@Fe-PCNFs retains 81.1% of its reference capacity after 3000 cycles. In comparison, δ-MnO_2_@PCNFs delivers a capacity retention of 47.1% after 3000 cycles.

**Figure S21** Optical image of δ-MnO_2_@Fe-PCNFs.

**Figure S22** a) The high-loading rate capacity at different current densities, b) high-loading long-term cycling performance at 1 A g^-1^ of δ-MnO_2_@Fe-PCNFs.

**Discussion**: At 0.2 A g^-1^, this high-loading electrode delivered an areal capacity of approximately 0.42 mAh cm^-2^. As the current density increased from 0.2 to 5 A g^-1^, the areal capacity gradually decreased, which is consistent with the enhanced ion-transport limitations and polarization commonly observed in thick electrodes. Nevertheless, an areal capacity of approximately 0.18 mAh cm^-2^ was still retained even at 5 A g^-1^. More importantly, during 1000 charge/discharge cycles, the high-loading electrode maintained a stable areal capacity of approximately 0.21-0.24 mAh cm^-2^ throughout cycling, while the Coulombic efficiency remained close to 100%. The limited capacity increase during the initial cycles can be attributed to gradual electrode activation and electrolyte infiltration within the thick electrode. Although the thicker electrode showed the expected mass-transport-related capacity decay at high current densities, the capacity recovered markedly when the current density was returned to a lower rate, and stable cycling performance was maintained over 1000 cycles. These results demonstrate that the Fe-regulated structure retains effective electrochemical activity and structural robustness even under increased mass loading.

**Figure S23** Long cycling performance of the flexible pouch cell of δ-MnO_2_@Fe-PCNFs//Zn at 1 A g^-1^.

**Table S1** Surface area and pore volume according to BET testing of δ-MnO_2_@Fe-PCNFs, δ-MnO_2_@PCNFs and PCNFs.

| Materials | Surface area (m^2^ g^-1^) | Pore volume (cm^3^ g^-1^) |
| --- | --- | --- |
| δ-MnO_2_@Fe-PCNFs | 14.57 | 0.06 |
| δ-MnO_2_@PCNFs | 20.39 | 0.08 |
| PCNFs | 41.04 | 0.13 |

**Table S2** Quantitative comparison of XRD-derived structural parameters for δ-MnO_2_@PCNFs and δ-MnO_2_@Fe-PCNFs.

| XRD feature | δ-MnO_2_@Fe-PCNFs  2θ, d | δ-MnO_2_@PCNFs  2θ, d | Δd (%) |
| --- | --- | --- | --- |
| (001) | 12.36°, 7.15Å | 12.56°, 7.04Å | +1.63 |
| (-111) | 36.97°, 2.43Å | 37.08°, 2.42Å | +0.27 |
| (020) | 65.70°, 1.42Å | 66.27°, 1.41Å | +0.71 |

**Table S3** The content of Mn and Fe in δ-MnO_2_@Fe-PCNFs and δ-MnO_2_@PCNFs.

| Materials | Mn | Fe |
| --- | --- | --- |
| δ-MnO_2_@Fe-PCNFs | 16.5 | 3.0 |
| δ-MnO_2_@PCNFs | 19.7 | 0 |

**Table S4** The EIS fitting results of δ-MnO_2_@Fe-PCNFs, δ-MnO_2_@PCNFs, Mn, Fe@PCNFs, and δ-MnO_2_.

| Material | R_S_ (Ω) | R_ct_ (Ω) |
| --- | --- | --- |
| δ-MnO_2_@Fe-PCNFs | 2.0 | 59.5 |
| δ-MnO_2_@PCNFs | 7.5 | 66.6 |
| Mn, Fe@PCNFs | 4.9 | 78.5 |
| δ-MnO_2_ | 5.8 | 60.8 |

**Table S5** Zinc-ion diffusion coefficients during discharge of δ-MnO_2_@Fe-PCNFs, δ-MnO_2_@PCNFs, Mn, Fe@PCNFs, and δ-MnO_2_.

| Material | $D_{Z\text{n}^{2+}}$ (cm^2^ s^-1^) |
| --- | --- |
| δ-MnO_2_@Fe-PCNFs | 9.7 × 10^-13^ |
| δ-MnO_2_@PCNFs | 4.6 × 10^-13^ |
| Mn, Fe@PCNFs | 1.7 × 10^-13^ |
| δ-MnO_2_ | 5.4 × 10^-13^ |

**Table S6** Comparison of long cycling performance of MnO_2_-based cathode materials for AZIBs.

| **Sample** | **Current density**  **(A g^-1^)** | **Cycle number** | **Capacity**  **(mAh g^-1^)** | **Capacity retention (%）** | **Ref.** |
| --- | --- | --- | --- | --- | --- |
| Cr-MnO_2_ | 1 | 300 | 113.87 | 65.8 | [6] |
| 3%Cr-MnO_2_ | 1 | 300 | 200 | 85.3 | [7] |
| V_0_-CMO | 1 | 700 | 54.3 | ~28.3 | [8] |
| Ag-MnO_2_ | 0.5 | 500 | 150 | 94.4 | [9] |
| V- MnO_2_/rGO | 1 | 1000 | 177.2 | 88.6 | [10] |
| TMA-MnO_2_ | 0.5 | 200 | 269 | 88 | [11] |
| K_0.28_MnO | 2 | 1000 | 100 | 95 | [12] |
| δ-CMO | 1 | 1000 | 225.3 | 77.8 | [13] |
| Se, P-MnO_2_ | 1 | 1000 | 232 | ~66.2 | [14] |
| Cu_2_-KMO | 1 | 1000 | 85.2 | 75.1 | [2] |
| K-δ-MnO_2_ | 2 | 1000 | 63.5 | 42.3 | [15] |
| **δ-MnO_2_@Fe-PCNFs** | **1** | **1000** | **243.64** | **/** | **This work** |
|  | **5** | **3000** | **59.58** | **81.1** |  |

**Reference**

1.	L. Li, Y. Jing, J. Zhang, et al., " Photocatalytic Degradation of NO by MnO_2_ Catalyst: The Decisive Relationship Between Crystal Phase, Morphology and Activity," *Journal of Hazardous Materials* 487, (2025): 137228. https://doi.org/10.1016/j.jhazmat.2025.137228.

2.	K. X. Ma, S. Y. Ge, R. R. Fu, et al., " Uncovering Se, P Co-doping effect in MnO_2_ toward High-performance Aqueous Zinc-ion Batteries," *Chemical Engineering Journal* 484, no. (2024): 149525. https://doi.org/10.1016/j.cej.2024.149525.

3.	S. J. Clark, M. D. Segall, C. J. Pickard, et al., "First Principles Methods Using CASTEP," *Zeitschrift für Kristallographie - Crystalline Materials* 220, no. 5 (2005): 567. https://doi.org/10.1524/zkri.220.5.567.65075.

4.	J. P. Perdew, K. Burke, M. Ernzerhof, "Generalized Gradient Approximation Made Simple," *Physical Review Letters* 77, no. 18 (1996): 3865. https://doi.org/10.1103/PhysRevLett.77.3865.

5.	D. Vanderbilt, " Soft Self-Consistent Pseudopotentials In A Generalized Eigenvalue Formalism," *Physical Review B* 41, no. 11 (1990): 7892. 10.1103/PhysRevB.41.7892.

6.	J. Hua, X. Wu, F. Xie, et al., " Cr-doping δ-MnO_2_ as the Cathode Material for Aqueous Zinc-ion Batteries," *Surfaces and Interfaces* 62, (2025): 106312. https://doi.org/10.1016/j.surfin.2025.106312.

7.	X. Ding, Y. Wen, C. Qing, et al., " Cr-induced Enhancement of Structural Stability in δ-MnO_2_ for Aqueous Zn-ion Batteries," *Journal of Alloys and Compounds* 986, no. (2024): 174041. https://doi.org/10.1016/j.jallcom.2024.174041.

8.	X. Chen, Z. Xu, S. Guo, et al., "Oxygen Vacancy-Rich Cobalt-Doped MnO_2_ Nanorods for Zn Ion Batteries," *ACS Applied Materials & Interfaces* 17, no. 8 (2025): 12074. https://doi.org/10.1021/acsami.4c19746.

9.	Y. Liao, C. Yang, Q. Xu, et al., in *Secondary* "Ag-Doping Effect on MnO_2_ Cathodes for Flexible Quasi-Solid-State Zinc-Ion Batteries," 8, no. 12 (2022): 267. https://doi.org/10.3390/batteries8120267.

10.	Y. Liu, Y. Wang, Y. Ma, et al., "Uniform Electric Fields-assisted MnO_2_/rGO Electrode for High-Performance Zinc-Ion Batteries," *Small* 21, no. 36 (2025): e04484. https://doi.org/10.1002/smll.202504484.

11.	A. N. Zhang, X. Zhang, H. N. Zhao, et al., " MnO_2_ Superstructure Cathode with Boosted Zinc Ion Intercalation for Aqueous Zinc Ion Batteries," *Journal of Colloid and Interface Science* 669, (2024): 723. https://doi.org/10.1016/j.jcis.2024.05.052.

12.	Y. D. Jiao, L. Q. Kang, J. Berry-Gair, et al., " Enabling Stable MnO_2_ Matrix for Aqueous Zinc-ion Battery Cathodes," *Journal of Materials Chemistry A* 8, no. 42 (2020): 22075. https://doi.org/10.1039/d0ta08638j.

13.	K. Li, Y. Liang, J. Wu, et al., " Cu^2+^ Intercalation Bolstering the Rate Capability of δ-MnO_2_ Cathode for Aqueous Zinc-ion Battery," *Journal of Energy Storage* 101, (2024): 113882. https://doi.org/10.1016/j.est.2024.113882.

14.	X. Hu, Y. Liao, M. Wu, et al., " Mesoporous copper-doped δ-MnO_2_ Superstructures to Enable High-performance Aqueous Zinc-ion Batteries," *Journal of Colloid and Interface Science* 674, (2024): 297. https://doi.org/10.1016/j.jcis.2024.06.152.

15.	Q. Xie, G. Cheng, T. Xue, et al., " Alkali Ions Pre-intercalation of δ-MnO_2_ Nanosheets for High-capacity and Stable Zn-ion Battery," *Materials Today Energy* 24, (2022): 100934. https://doi.org/10.1016/j.mtener.2021.100934.
